# Advances in Anterior Cruciate Ligament Injury, Reconstruction and Rehabilitation

**DOI:** 10.3390/medicina60010136

**Published:** 2024-01-11

**Authors:** Adrian Todor

**Affiliations:** Department of Orthopaedics and Traumatology, “Iuliu Hatieganu” University of Medicine and Pharmacy, No. 8 Victor Babes Street, 400000 Cluj-Napoca, Romania; adi.todor@yahoo.com or adrian.todor@umfcluj.ro

Another Special Issue dedicated to the anterior cruciate ligament (ACL) of the knee joint. Why, may you ask? Looking at the literature one could find thousands of papers regarding ACL injury, treatment and rehabilitation. However, it is also obvious from looking at the literature that there is much room for improvement.

ACL tears are one of the most frequently encountered pathologies for the orthopaedic surgeon, with more than 100,000 ACL surgeries being performed per year in the USA [1]. Additionally, the incidence of ACL tears has been reported to be increasing constantly through the years, especially affecting the young population and adolescents [2]. This fact indicates that the injury prevention programs that have been implemented until now have been unsuccessful in achieving their goals [3].

ACL reconstruction is still the gold-standard treatment for this injury in the athletic population; however, failure has been reported to be as high as 24% [3] and only 50% to 65% of recreational athletes return to their preinjury level of sports [4].

Clearly, we must do better. Whether it is through injury prevention, improved treatment strategies and rehabilitation or a combination, we need to improve our results. This Special Issue was opened with this idea in mind and has published a total of six original papers, adding to the existing knowledge base on ACL and related topics.

In the community of orthopaedic surgeons, much attention has been directed at improving results through changes in surgical techniques such as graft selection [5], fixation and tunnel placement [6] and single- vs. double-bundle techniques [7].

Regarding graft fixation, the article by Kong Y et al. [8] verified whether the position of the tibial compression screw impacts postoperative tunnel enlargement rates and clinical outcomes. Interestingly, the authors found that posterior interference screw insertion leads to less tunnel enlargement at the tibial tunnel near the articular surface of the tibia, but with no significant differences in clinical outcomes. Still, to create better conditions for graft healing and revision, posterior screw insertion was recommended by the authors.

In recent years, research has also focused on the role of the anterolateral ligament (ALL) and the lateral extra-articular tenodesis (LAT) in improving the outcomes of ACL reconstruction [4,9] and the primary repair of the ACL [10,11].

It is worth mentioning the article by Cheng YH et al. [12] published in this Special Issue investigating the prognosis of combined ACL and ALL reconstruction. The authors retrospectively reviewed patients who underwent combined ACL and ALL reconstruction and had a minimum follow-up of 2 years. Their results indicated that patients displayed no graft tear or low functional knee instability with this combined procedure. This is in accordance with other studies showing reductions in re-tear rates of ACL reconstruction when a LAT procedure is added [13,14].

Regarding rehabilitation, it is well known and universally agreed that physical therapy plays a critical role in the successful recovery of both surgically and non-surgically managed patients with ACL tears. Recent strategies suggest a more individualized approach to the rehabilitation of ACL-injured patients with protocols modifiable to patients’ specific needs and pace of progress [15].

In conclusion, although significant research has been performed, and continues to be added to the literature daily, progress still needs to be made regarding ACL-related pathology in order to hopefully improve the outcomes of patients suffering from ACL tears.
